# The Effect of Antenatal Care Service Utilization on Postnatal Care Service Utilization: A Systematic Review and Meta-analysis Study

**DOI:** 10.1155/2020/7363242

**Published:** 2020-09-22

**Authors:** Alehegn Bishaw Geremew, Moges Muluneh Boke, Ayenew Engida Yismaw

**Affiliations:** ^1^Department of Reproductive Health, Institute of Public Health, College of Medicine and Health Sciences, University of Gondar, Ethiopia; ^2^Department of Clinical Midwifery, School of Midwifery, College of Medicine and Health Sciences, University of Gondar, Ethiopia

## Abstract

**Introduction:**

Reduction of maternal and neonatal morbidity and mortality has continued to be a challenge in developing countries. The majority of maternal and neonatal mortality occurred during the early postpartum period. This is mostly due to low postnatal care service utilization. There is a discrepancy of evidence on the effect status of antenatal care on the improvement of postnatal care service utilization. Therefore, this review study is aimed at estimating the pooled effect of antenatal care on postnatal care service utilization.

**Methods:**

We searched from PubMed and Cochrane library database, Google Scholar, and Google. Initially, we found 265 articles; after duplication was removed and screened by the relevance of the titles and abstracts, 36 studies were considered for assessment of eligibility. Finally, 14 articles passed the inclusion and exclusion criteria and are included in the meta-analysis. Study quality assessment was done using Janna Briggs Institute (JBI) critical appraisal tools. The main information was extracted from each study. Heterogeneity of studies was assessed using *I*^2^ = 70% and more considered having high heterogeneity. The publication bias was checked using funnel plot and big test. Meta-analysis using a random effect model was conducted. A forest plot was used to show the estimated size effect of odds ratio with a 95% confidence interval.

**Results:**

A total of 14 articles were included with 15,765 participants for synthesis and meta-analysis. We found that a pooled estimate of women who had antenatal care was 1.53 times more likely to have postnatal care compared with those who had no antenatal care (AOR = 1.53, 95% CI 1.38-1.70, *I*^2^ = 0%).

**Conclusions:**

This review results revealed a low utilization of postnatal care service. Antenatal care service utilization has a positive effect on postnatal care service utilization. Policymakers and programmers better considered more antenatal care service use as one strategy of enhancing the utilization of postnatal care service.

## 1. Introduction

Maternal mortality remains the major challenge worldwide. According to WHO report globally, an estimated 303,000 maternal death occurred in 2015; developing regions account 99% of global the estimates [1]. Every day, about 830 women died due to the complication of pregnancy and childbirth. The primary cause of maternal morbidity and death is haemorrhage, high blood pressure, and sepsis [2]. Most maternal and infant death occurs in the first one month after birth; almost half of postnatal maternal death occurs within the first 24 hours [3]. Globally, neonatal mortality reduction is slower compared with under-five mortality reduction, in which neonatal health highly intertwined with maternal health service use [4].

Among different maternal health services, postnatal care service is one of the crucial care to reduce both maternal and neonatal mortality. The postpartum period is vital for early detection of both maternal and new born health problems, counselling on physiological recovery, follow-up for the continuum of care, and providing family planning service [5]. WHO recommended postnatal care in the first 24-48 hours to all mothers and babies regardless of where the birth occurred and provide every mother and new born a total of four postnatal visits [6]. Assessments could be done at each postnatal contact regardless of the place of delivery such as urinary incontinence, bowel function, healing of any perennial wound, headache fatigue, perennial pain, uterine tenderness, and lochia [7].

From all maternal health services, postnatal care service utilization remained low in developing countries [8]. For example according to Ethiopian Demography and Health Survey (EDHS) 2016 in Ethiopia, there is a high discrepancy between the proportion of antenatal care utilization (62%) and postnatal care service utilization within the first two days of delivery (17%) [9]. In another study in Ethiopia, the prevalence of PNC within six weeks of postpartum period was 31.7%, and ANC is statistically associated with PNC [10].

Countries have been implementing different strategies targeted to improve maternal health service use including postnatal care service. Although antenatal care service utilization has been increasing, enhancing postnatal care service use remained a challenge and evidence of antenatal care use association with postnatal care varies among studies.

Therefore, we conducted this systematic review and meta-analysis to assess pooled evidence on the effect of antenatal care service utilization on postnatal care service utilization.

## 2. Methods

### 2.1. Source and Search Strategies

We made search from PubMed and Cochrane library database and Google Scholar and Google grey literatures for relevance in medical and all fields with subheading with keywords. We focused on articles published in English until September 2017.We have done article and grey literature search from September 2017 to October 2017. Our search strategies combined terms related to antenatal care and postnatal care in East African countries. We had search also grey literatures using keywords without full search protocol. Our full search protocol is available (see supplemental file 1).

Endnote referencing software was used to collect the articles and acknowledge the authors through citations. We screened titles and abstracts for relevance; duplicates were removed, and then we analysed the full-text potentially relevant articles assessed for inclusion and exclusion criteria. For African content division geographically, according to the united nation statistics division schemes, for the geographic region of East Africa, the countries are named Burundi, Comoros, Djibouti, Eritrea, Ethiopia, Kenya, Madagascar, Malawi, Mozambique, Rwanda, Seychelles, Somalia Tanzania, Uganda, Zambia, Zimbabwe, South Sudan, and Sudan [11].

### 2.2. Inclusion and Exclusion Criteria

The following inclusion criteria were used: Women had at least one follow-up within 42 days after delivery during the index child birth, study population maximum of five years after index childbirth, and study design (experimental, cohort study, case-control, and cross-sectional study). Articles published before 2010, studies that did not consider antenatal care as determinants of postnatal care, outcome measured having full postnatal care, postnatal care use measured with in the first 6 hours of delivery, and qualitative studies are used as exclusion criteria.

The outcome of interest was postnatal care service utilization at least one within 42 days after delivery, whereas antenatal care service utilization was assessed as a determinant of postnatal care service utilization.

### 2.3. Quality Assessment

The scientific quality of each eligible article was assessed using Janna Briggs Institute (JBI) critical appraisal tools [12]. Two individuals working independently assessed the scientific quality of each eligible study. For the eligible cross-sectional study, the parameter was considered during the quality assessment were appropriateness of sampling procedure, adequacy of sample size, outcome measurement, and response rate are done. The total scoring system comprised of 9 criteria, and different quality score recorded for each eligible article. There was a high level of agreement between the reviewers about the information extracted from each article. The discrepancy on the assessment of the scientific quality of the articles was resolved through discussion with the third author.

### 2.4. Data Extraction

Data were extracted by two reviewers using Excel sheet standardized form developed by all authors, and the information extracted from each article were as follows: first author name, country, year of publication, study design and setting, measurement of postnatal care, sample size, number of women use PNC, antenatal care status of participants, antenatal care status of women had postnatal care use, and adjusted odds ratio/relative risk with confidence interval. For incomplete data, an attempt to contact the corresponding author was made by email. However, for a single article, the antenatal care status of participants and antenatal care status of the outcome variable was not obtained. As a result, we have used the adjusted odds ratio of this particular article to estimate the logarithm of the odds ratio and standard error. The difference in the extracted information between the two reviewers was solved by a joint discussion with the third individual.

### 2.5. Data Synthesis and Analysis

The qualitative data synthesis was made considering the study setting, the proportion of postnatal care, and the statistical significant status of each included articles. After the generation of the required parameter, the extracted data were transferred from Excel to Stata version 14 for future analysis. We pooled the proportion of postnatal care service utilization and the measure of association (odds ratio/relative risk) with a 95% confidence interval for antenatal care service utilization as determinants of postnatal care service utilization. To obtain the pooled effect size of antenatal care, a meta-analysis using a random-effect model was done. The heterogeneity of the study was assessed using *I*^2^ test statistics with a value of *I*^2^ = 25%, 50%, and 70 represented low, moderate, and high heterogeneity, respectively, considered substantial heterogeneity [13]. *I*^2^ is measure of the proportion of the total variation in the study estimate that is due to heterogeneity. The possibility of publication bias was assessed using a funnel plot for its asymmetry objectively, and the Egger test and big test if *p* value < 0.05 to consider that there is evidence of publication bias [14].

### 2.6. Reporting

The results are presented using a text, table, and forest plot with effect measure and odds ratio with 95% confidence interval. The results of this review have been reported according to the preferred reporting item for systematic review and meta-analysis (PRISMA statement) guideline [15] (see supplemental file 2).

## 3. Main Text

### 3.1. Search Results and Characteristics of the Studies

**Figure 1:**
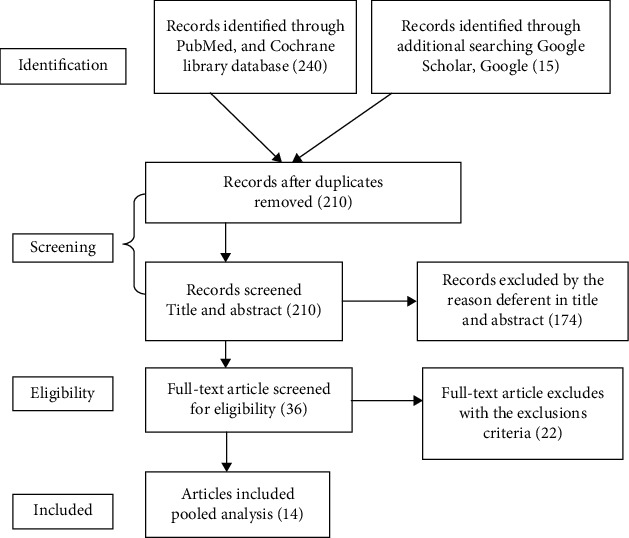
PRISMA flow diagram summarizing the literature search.

Our initial search produced 265 articles which were of potential interest. After removing duplicates, screening abstract, and full-text review, we got 14 studies that met our inclusion criteria [10, 16–27] (Figure 1). All studies included for this review were published between 2011 and 2017. Fourteen studies with a total of 15,765 participants were included in the review. The sample size of included studies ranged from 399 to 3,970 participants. The data extracted from each study were included in the meta-analysis to the pooled proportion of postnatal care service use and adjusted odds ratio (AOR) of antenatal care. The reviewed studies comprised of one longitudinal follow-up and thirteen cross-sectional studies, and study settings of the 14 studies passed and were included in synthesis, 8 of them were primarily conducted in Ethiopia [10, 16, 17, 19, 22, 23, 25, 28], two from Kenya [18, 24], one each from Tanzania [26], Rwanda [27], South Sudan [21], and Zambia [20]. All the included studies pass the quality assessment ≥ 6/9 score. Ten of the studies deserved 6-7/9and four of the articles scored 8/9 scientific quality according to the JBI quality appraisal checklist.

### 3.2. Postnatal Care Service Utilization

The proportion of postnatal care service utilization in the reviewed articles ranged from 11.4% to 66.8%. Among the reviewed articles, the proportion of postnatal care service utilization reported in four studies were more than 50% and out of studies with PNC more than 50%, three studies are from Ethiopia [16, 23, 28] and one from Zambia [20]. In findings reported within the six studies, the proportion of PNC ranges from 25% to 50% [10, 17–19, 22, 24]. However, within four studies, the proportion of PNC was less than one-fourth [21, 25–27] (Table 1).

### 3.3. Effects of Antenatal Care on the Utilization of PNC Service

Our assessment of studies indicated that variation in PNC service utilization is according to ANC service utilization status during the recent pregnancy. Findings from nine studies indicated that ANC service utilization had a statistically significant effect on PNC service utilization [10, 16–18, 20, 22, 24, 25, 28]. However, findings reported from five studies antenatal care use was not associated with postnatal care service utilization [19, 21, 23, 26, 27](Table 1).

**Figure 2:**
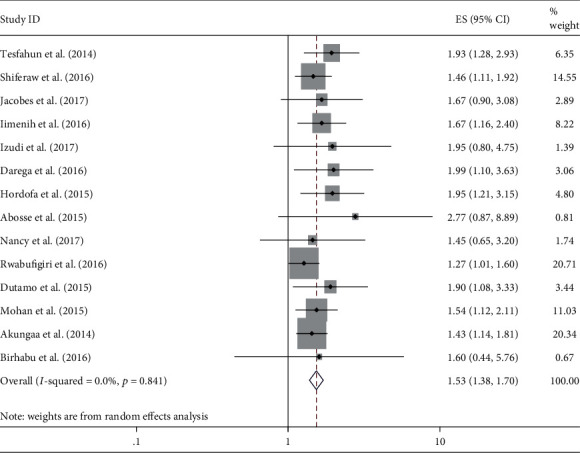
Forest plot shows the effect of antenatal care use on postnatal care service utilization. The midpoint and the length of each line segment indicate odds ratio and 95% confidence interval, respectively, whereas the diamond shape indicate the overall pooled estimate of odds ratio for the fourteen studies.

The meta-analysis results revealed that the pooled estimate odds ratio of antenatal care found was statistically associated with postnatal care service use (OR = 1.53, 95% CI: 1.38-1.70, *I*^2^ = 0.0%, *p* = 0.841). The *I*^2^ value indicated that there is no heterogeneity between studies. Women who had antenatal care use 1.53 times were more likely to have postnatal care service use compared with those who had no antenatal care during the index pregnancy (Figure 2). We have checked publication bias using a funnel plot (see supplemental file 3), and Egger's test (*p* value = 0.21) indicates that there was no statistical evidence of publication bias.

## 4. Discussions

Antenatal care is an entry point of pregnant women to the health care system, and it is one of the interventions to reduce maternal and neonatal morbidity and mortality [29]. Antenatal care provides important health care functions including screening, diagnosis, and treatment of health problems, health promotion, and counselling on continuity of ANC, delivery care, and postnatal care [30]. The country like Ethiopia and other African countries implemented the World Health Organization (WHO) recommendation of focusing on antenatal care (FANC) having four visits for every pregnant woman. Every childbirth woman is recommended to have three postnatal care regardless of the place of delivery [31]. However, the need for care after childbirth is less recognized by women in developing countries [32].

Finding in this review, the pooled estimates of antenatal care utilization are statistically associated with postnatal care service use. Women who had antenatal care 1.53 times are more likely to have postnatal care than women who have no antenatal care (AOR = 1.53, 95% CI 1.38-1.70, *I*^2^ = 0%) This finding is supported by systematic analysis and meta-analysis and EDHS future analysis studies conducted in Ethiopia [33–35] and meta-analysis study done in low-and middle-income countries [36]; other studies conducted in India [37, 38], Ghana [39], Nepal, Tanzania [40], Nigeria [41]; and a community-based interventional study in Ethiopia [42]. This might be due to those women having access to information on the complication that can happen during the postpartum period, knowledgeable on postpartum danger sign, and aware about complication redness plan including where to go when the problem happens [43, 44].

## 5. Limitations

Articles were not found from some East African countries; this might affect the representativeness of the pooled estimate for the whole East African countries; we, the authors, tried to consider articles from each country but have not found a study passed the screening and fulfill the inclusion criteria. This review did not answer why postnatal care is the lowest compared to another maternal health service and why women who have antenatal care follow-up fails to receive postnatal care.

## 6. Conclusions and Recommendations

Our meta-analysis findings revealed that the postnatal care service utilization is low, despite postnatal care being a crucial care to prevent both maternal and neonatal morbidity and mortality. Antenatal care service utilization affects postnatal care service utilization. Policymakers and programmers better consider more antenatal care service use as one strategy of enhancing the utilization of postnatal care service. Providing information about the importance of postnatal care during antenatal care service by provider may be the best mechanism to improve the low PNC service utilization.

## Figures and Tables

**Table 1:** Characteristics of studies included in a systematic review.

First author/publication year	Country	Study design	Sample size	PNC	ANC	PNC		Key findings
						Yes	No	
Tesfahun et al./2014 [16]^∗^	Ethiopia	Cross-sectional	820	548	Yes	507	198	PNC proportion (66.8%), AOR 2.36, 95% CI 1.31-4.23
					No	41	74	
Shiferaw et al./2016 [17]^∗∗^	Ethiopia	Prospective follow-up	1,201	376	Yes	288	495	PNC proportion (31.3%), AOR 2.77, 95% CI 2.12-3.61
					No	88	362	
Jacobes et al./2017 [20]^∗^	Zambia	Cross-sectional	551	355	Yes	137	13	PNC proportion (64.4%), AOR 2.42, 95% CI 1.43-3.62
					No	289	89	
Limenih et al./2016 [19]^∗^	Ethiopia	Cross-sectional	588	197	Yes	138	163	PNC proportion (33.5%), AOR 1.01, 95% CI 0.54-1.91
					No	59	228	
Izudi et al./2017 [21]^∗^	South Sudan	Cross-sectional	385	44	Yes	38	196	PNC proportion (11.4%), ANC have no association with PNC
					No	6	145	
Darega et al./2016 [10]^∗^	Ethiopia	Cross-sectional	703	223	Yes	210	169	PNC proportion (31.7%), AOR 4.95, 95% CI 2.50-9.80
					No	13	111	
Hordofa et al./2015 [22]^∗^	Ethiopia	Cross-sectional	736	256	Yes	234	333	PNC proportion (34.8%), AOR 4.9, 95% CI 2.91-7.57
					No	22	147	
Abosse et al./2015 [25]^∗∗^	Ethiopia	Cross-sectional	691	157	Yes	152	442	PNC proportion (22.7%), AOR 0.148, 95% CI 0.039-0.56
					No	3	92	
Njoka et al./2017 [18]^∗^	Kenya	Cross-sectional	399	180	Yes	171	195	PNC proportion (45.1%), Chi square (*χ*^2^) 4.62, *p* value 0.03
					No	9	24	
Rwabufigiri et al./2016 [27]^∗∗^	Rwanda	Cross-sectional	2,748	351	Yes	145	691	PNC proportion (12.8%), AOR1.18, 95% CI 0.91-1.63
					No	206	1698	
Dutamo et al./2015 [23]^∗^	Ethiopia	Cross-sectional	623	320	Yes	303	243	PNC proportion (51.4%), AOR 1.2, 95% CI 0.4-3.5
					No	17	60	
Mohan et al./2015 [26]^∗^	Tanzania	Cross-sectional	1,931	437	—	—	—	PNC proportion (22.6%), AOR 2.71, 95% CI 0.7-6.3
					—	—	—	
Akungaa et al./2014 [24]^∗^	Kenya	Cross-sectional	3,970	1,882	Yes	1770	1830	PNC proportion 10.4%, AOR 1.89, 95% CI 1.23-2.9
					No	110	261	
Birhanu e tal/2016 [28]∗	Ethiopia	Cross sectional	422	277	Yes	273	139	PNC 65.6%, AOR 4.6, 95% CI 1.0-7.8
					No	4	6	

Abbreviation: ANC: antenatal care; PNC: postnatal care. ^∗^Quality 6-7/9. ^∗∗^Quality 8-9/9.

## Data Availability

The dataset supporting the conclusion of this article is included within the manuscript.

## Abbreviations

ANC: Antenatal care
FANC: Focused antenatal care
PNC: Postnatal care
WHO: World Health Organization
DHS: Demographic Health Survey.

## Supplementary Materials

Supplementary 1 Supplementary material 1: a full searching protocol on the effect of antenatal care use on postnatal care service utilization in East Africa countries.

Supplementary 2 Supplementary material 1: the PRISMA 2009 Checklist of effect of antenatal care service utilization on postnatal care service utilization.

Supplementary 3 Supplementary material 1: funnel plot without adjustment for publication bias, log, or of odds ratio in the x axis and standard error of log odds ratio in *Y* axis.
