# Supplementary material for: The Effect of Antenatal Care Service Utilization on Postnatal Care Service Utilization: A Systematic Review and Meta-analysis Study
Source: J Pregnancy. 2020 Sep 22;2020:7363242. doi: 10.1155/2020/7363242 (PMC7528140; doi:10.1155/2020/7363242)
Supplement: Supplementary 1 — Supplementary material 1: a full searching protocol on the effect of antenatal care use on postnatal care service utilization in East Africa countries. [file 7363242.f1.docx]

Full search protocol

ANC[All Fields] OR "prenatal care"[MeSH Terms] OR ("prenatal care"[MeSH Terms] OR ("prenatal care"[All Fields] AND "care"[All Fields]) OR "prenatal care"[All Fields] OR ("antenatal"[All Fields] AND "care"[All Fields]) OR "antenatal care"[All Fields]) OR FANC[All Fields] AND PNC[All Fields] OR PNC[All Fields] OR "postnatal care"[MeSH Terms] OR ("postnatal care"[MeSH Terms] OR ("postnatal"[All Fields] AND "care"[All Fields]) OR "postnatal care"[All Fields]) AND ("Africa, eastern"[MeSH Terms] OR ("Africa"[All Fields] AND "eastern"[All Fields]) OR "eastern Africa"[All Fields] OR ("east"[All Fields] AND "Africa"[All Fields]) OR "east Africa"[All Fields]).
