# Supplementary material for: The Effect of Antenatal Care Service Utilization on Postnatal Care Service Utilization: A Systematic Review and Meta-analysis Study
Source: J Pregnancy. 2020 Sep 22;2020:7363242. doi: 10.1155/2020/7363242 (PMC7528140; doi:10.1155/2020/7363242)
Supplement: Supplementary 3 — Supplementary material 1: funnel plot without adjustment for publication bias, log, or of odds ratio in the x axis and standard error of log odds ratio in Y axis. [file 7363242.f3.docx]

Supplementary material 3

Funnel plot for publication bias, Logor represented in the x-axis and standard error of log or in the y-axis
